# The Toxicokinetics, Excretion Patterns, and Milk Transmission of Ochratoxin A in Lactating Sows

**DOI:** 10.3390/toxins16030128

**Published:** 2024-03-01

**Authors:** Qiufeng Zhu, Honglei Qu, Ruifen Kang, Yunduo Zheng, Qiuying Guo, Shimeng Huang, Lihong Zhao, Qiugang Ma

**Affiliations:** 1 State Key Laboratory of Animal Nutrition and Feeding, College of Animal Science and Technology, China Agricultural University, Beijing 100193, China; qiufengzhizhou@163.com (Q.Z.); leihong_qu@163.com (H.Q.); ruifenkang@cau.edu.cn (R.K.); zhengyunduo@cau.edu.cn (Y.Z.); guoqiuying2001@163.com (Q.G.); shimengh@cau.edu.cn (S.H.); zhaolihongcau@cau.edu.cn (L.Z.); 2 Laboratory of Feedgrain Safety and Healthy Poultry Farming, Beijing Jingwa Agricultural Science and Technology Innovation Center, Beijing 100193, China

**Keywords:** Ochratoxin A, toxicokinetics study, lactating sows, milk

## Abstract

Ochratoxin A (OTA), a mycotoxin commonly found in feedstuffs, is known for its detrimental effects on the kidneys and liver, posing significant health risks to animals and humans. This study investigated the toxicokinetics, excretion patterns, and milk transmission of Ochratoxin A (OTA) in lactating sows. The sows were administered a single oral dose of 500 μg/kg BW (body weight), followed by the systematic sampling of plasma, feces, urine, and milk. Plasma samples were collected at 0, 5, 15, and 30 min, and 1, 2, 3, 6, 9, 12, 24, 48, 72, 88, 96, and 120 h post administration. Feces samples were collected at 6 h intervals for the first 12 h, then at 12 h intervals until 120 h, while urine samples were collected at 6 h intervals up to 120 h. Milk samples were collected at 0, 6, 12, 24, 36, 48, 72, 96, and 120 h. The concentration of OTA and its primary metabolite OTα were quantitatively analyzed using ultra-performance liquid chromatography tandem mass spectrometry (UPLC-MS/MS). The results revealed that the peak plasma concentrations of OTA (920.25 ± 88.46 μg/L) were observed at 9 h following administration. The terminal elimination half-life was recorded at 78.47 ± 7.68 h, with a volume of distribution of 0.16 ± 0.003 L/kg. Moreover, this study documented the excretion of OTA and OTα across a span of 120 h, revealing that feces and urine accounted for 18.70 ± 0.04% and 8.40 ± 0.002% of the total intake amounts, respectively (calculated based on substance amounts). Furthermore, this experiment detected OTA residues in the milk of lactating sows, with the milk-to-plasma (M/P) ratio initially increasing from 0.06 to 0.46 within the first 24 h following OTA ingestion. These findings offer an exhaustive temporal analysis of OTA’s toxicokinetics in lactating sows, emphasizing its pervasive distribution and elimination through various bodily excreta.

## 1. Introduction

Ochratoxins, particularly Ochratoxin A (OTA), are a group of mycotoxins produced by several fungi such as *Aspergillus ochraceus* and *Penicillium verrucosum*. These toxins are pervasive in agricultural commodities like maize [1], wheat [1], barley [1], rice, and rice-products [2], as well as in dried fruit [3], coffee [3,4], cocoa [3], animal feed [1], pork [5], and ewe’s milk [6], representing a significant health hazard to both humans and animals [7,8,9]. OTA is recognized for its extensive harmful effects across various species, affecting zootechnical performance (e.g., body weight gain, feed/gain ratio, etc.) [1,10], leading to reductions in average daily feed intake (ADFI) and egg weight (EW) in laying hens [11], as well as inducing immunosuppression and inflammation [12,13].

On a molecular level, OTA interferes with phenylalanine in reactions catalyzed by phenylalanine-tRNA synthetase and phenylalanine-hydroxylase, while also exacerbating lipid peroxidation [14,15]. This dual action disrupts cellular mechanisms and promotes oxidative stress, causing organ damage, particularly in the liver and kidneys, which are the main sites of OTA accumulation and toxicity [16,17].

OTA’s toxicokinetics have been widely studied in various animal species, with administration typically through the oral route or via injection. This thorough investigation spans a diverse array of species, including growing pigs [18], rabbits [18], chickens [18], rats [19], monkeys [20], dairy ewes [6], Atlantic salmon [21], and donkeys [22]. There is a marked variability in sensitivity to OTA among different species, with livestock and poultry, particularly pigs, showing considerable susceptibility due to a higher affinity for serum proteins [10,18]. The efficient absorption and slow elimination of OTA contribute to its persistence in animals [22], raising concerns about its accumulation in animal products and subsequent transfer to humans through consumption, thereby affecting human health.

Despite existing studies on OTA’s toxicokinetics in growing pigs [18] and its effects on nursery pigs [23], research on lactating sows is absent. This gap is significant considering the unique physiological and metabolic changes during lactation, which can affect the detoxification and excretion of toxins like OTA. Moreover, the potential of OTA excretion through milk presents an indirect risk to piglets.

Given these considerations, the primary objective of this study is to explore the toxicokinetics of OTA in lactating sows and to examine the potential transmission of OTA through sow’s milk.

## 2. Results

### 2.1. Validation Parameters

**Figure 1:**
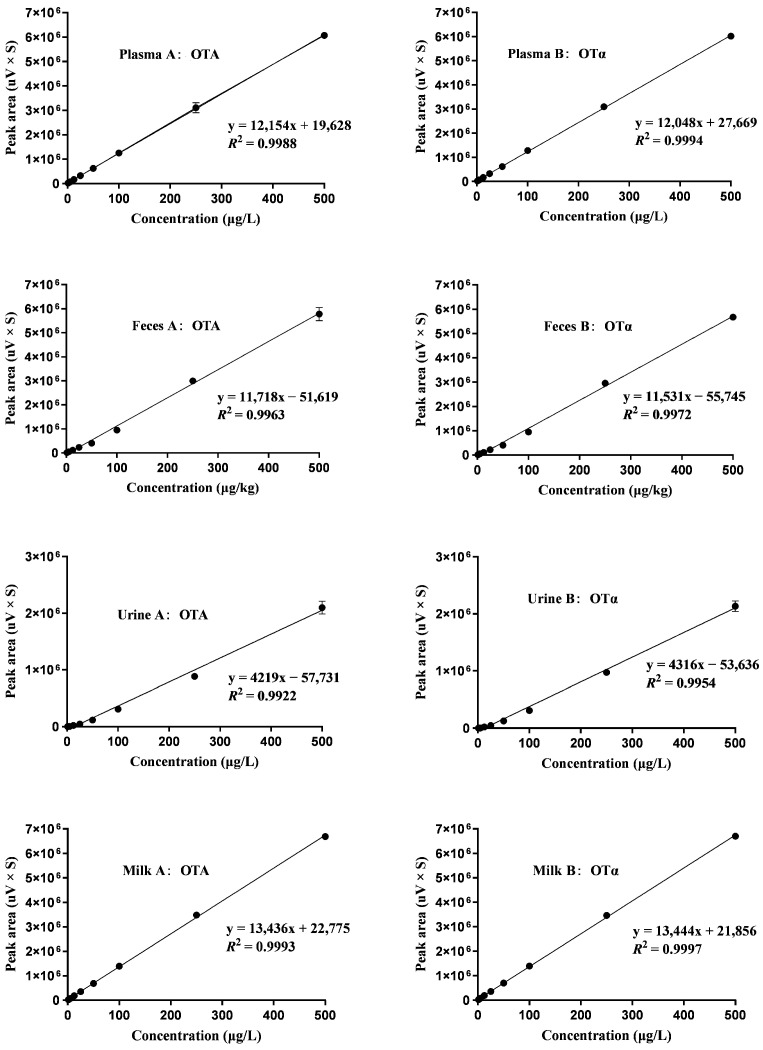
Calibration curves for spiked samples, *n* = 3. The curves display data for concentrations of 1.25, 2.5, 5, 12.5, 25, 50, 100, 250, and 500 μg/L in plasma (A-B), feces (A-B), urine (A-B), and milk (A-B).

The calibration curve shown in Figure 1 for OTA and its metabolite OTα demonstrated a linear relationship across a concentration spectrum of 1.25–500 μg/L. High *R*^2^ values were obtained for OTA and OTα in plasma (*R*^2^ = 0.9988, 0.9994), feces (*R*^2^ = 0.9963, 0.9972), urine (*R*^2^ = 0.9922, 0.9954), and milk (*R*^2^ = 0.9993, 0.9997), respectively. The limits of detection (LOD) and quantification (LOQ) for OTA and OTα were determined as follows: in plasma (LOD = 0.2 μg/L, LOQ = 0.6 μg/L), feces (LOD = 0.4 μg/kg, LOQ = 1.2 μg/kg), urine (LOD = 0.2 μg/L, LOQ = 1.0 μg/L), and milk (LOD = 0.2 μg/L, LOQ = 0.6 μg/L), as summarized in Table 1. The mean recovery rates of OTA were in the range of 84.25–87.03%, 82.50–85.50%, 82.30–90.68%, and 92.78–96.65% in plasma, feces, urine, and milk, while those of OTα were 85.23–86.95%, 83.60–87.20%, 84.50–92.35%, and 93.60–85.20%, respectively (Table 2).

### 2.2. Plasma Toxicokinetics Parameters of OTA and OTα in Lactating Sows

Subsequent to a single oral intake of OTA at 500 μg/kg BW, the toxin was assimilated into the blood circulation of lactating sows with an average body weight of 186.25 ± 10.30 kg. Plasma samples were subsequently collected to detect OTA and OTα levels. As indicated in Table 3, at 9.00 ± 0.00 h (Tmax) after administration, OTA reached its peak concentration (Cmax) in plasma at 920.25 ± 88.46 μg/L. The elimination half-life of OTA was calculated to be 78.47 ± 7.68 h, the area under the plasma concentration–time curve (AUC) was determined to be 69.50 ± 7.90 μg·mL^−1^·h, the mean residence time (MRT) was 107.78 ± 9.33 h, the total plasma clearance (Cl) was 0.0014 ± 0.00 L·kg BW^−1^·h^–1^, and the volume of distribution (Vd) was 0.16 ± 0.003 L/kg BW.

OTα reached its peak concentration (Cmax, 3.45 ± 0.07 μg/L) in plasma at 12.00 ± 0.00 h after administration. The elimination half-life of OTα was calculated to be 33.61 ± 12.13 h, the area under the plasma concentration–time curve (AUC) was determined to be 0.21 ± 0.01 μg·mL^−1^, the mean residence time (MRT) was 51.91 ± 11.98 h, the total plasma clearance (Cl) was 0.44 ± 0.02 L·kg BW^−1^·h^–1^, and the volume of distribution (Vd) was 26.18 ± 13.36 L/kg BW.

### 2.3. Plasma Concentration of OTA and OTα

**Figure 2:**
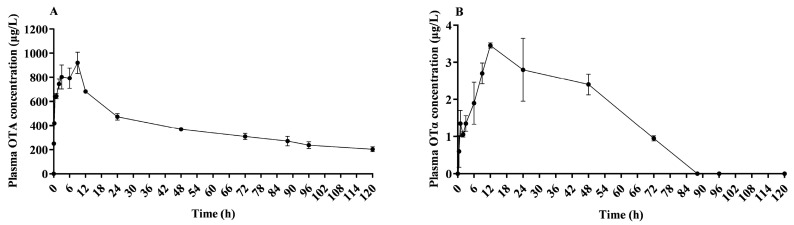
Plasma mean concentration–time profile of OTA and OTα in lactating sows. This plot illustrates the variation in plasma mean concentration of OTA (**A**) and OTα (**B**) over time, following an oral dose of 500 μg/kg BW in lactating sows; *n* = 4.

As depicted in Figure 2A, OTA was initially detected in plasma just 5 min after a single oral administration. The concentration progressively increased until the peak concentration was reached at 9.00 ± 0.00 h, and then subsequently declined. The highest recorded plasma concentration of OTA was 920.25 ± 88.46 μg/L. As shown in Figure 2B, only trace levels of OTα were detected in plasma. OTα was initially detected in plasma 1 h after a single oral dose. The concentration progressively increased, peaking at 12.00 ± 0.00 h, and then declined. The highest recorded plasma concentration of OTα was 3.45 ± 0.07 μg/L.

### 2.4. Excretion of OTA and OTα through Feces, Urine, and Milk

**Figure 3:**
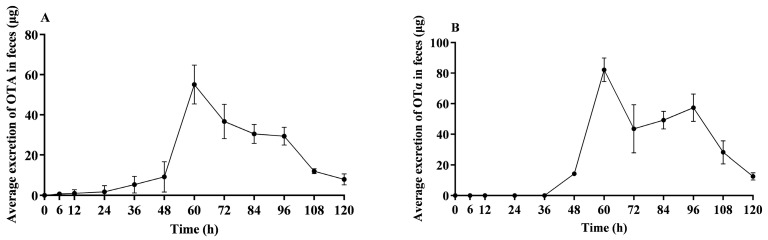
Average OTA and OTα excretion in feces of lactating sows. This figure illustrates the average excretion of unconjugated OTA (**A**) and OTα (**B**) in feces over time in lactating sows following a single oral dose of 500 μg/kg BW; *n* = 4.

Figure 3A illustrates that within the initial 24 h post OTA administration, OTA fecal elimination remained relatively low. A significant increase in OTA fecal elimination was observed from 24 to 60 h, with peak excretion being reached at 60 h. Following this apex, the quantity of eliminated OTA through feces began to decrease, ultimately resulting in minimal detectable levels by 120 h.

Figure 3B illustrates that within the initial 48 h post OTA administration, OTα fecal elimination remained relatively low. The excretion progressively increased, peaking at 60 h, and then declined, ultimately resulting in only minimal detectable levels in feces by 120 h.

**Figure 4:**
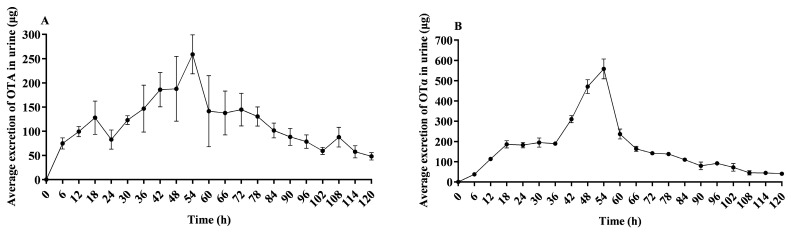
Average OTA and OTα excretion in urine of lactating sows. This figure presents the average OTA (**A**) and OTα (**B**) excretion in urine over time in lactating sows following a single oral dose of 500 μg/kg BW; *n* = 4.

Figure 4A demonstrates the elimination of OTA in urine of lactating sows post administration. The peak of OTA elimination was observed at 54 h, after which there was a decreasing trend in OTA clearance. Only trace amounts of OTA were detectable in urine up to 120 h. Figure 4B demonstrates the elimination of OTα in urine of lactating sows post administration. The peak of OTα elimination was observed at 54 h, after which there was a decreasing trend in OTA clearance. Only trace amounts of OTα were detectable in urine up to 120 h.

**Figure 5:**
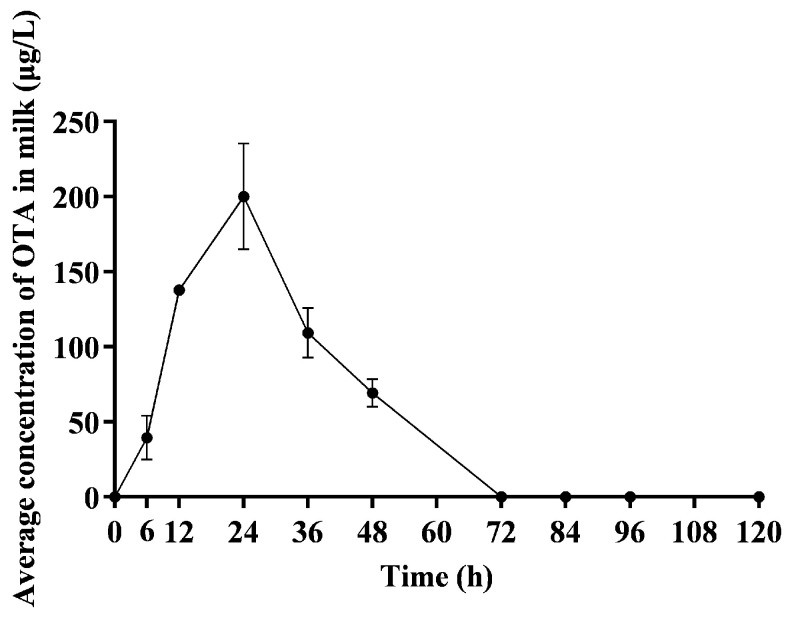
Average OTA concentration in milk of lactating sows. This figure shows the average concentration of OTA in milk over time in lactating sows after receiving a single oral dose of 500 μg/kg BW of OTA, *n* = 4.

As shown in Figure 5, only OTA was detected and its concentration in milk was initially low during the first 6 h following administration. However, a marked increase in OTA concentration was observed between 6 and 24 h, culminating in peak concentrations at 24 h. After reaching this peak, the concentration of OTA gradually declined, with only trace amounts detectable in the milk by 72 h.

As shown in Table 4, lactating sows had a total OTA intake of 93.12 ± 5.15 mg. Calculated based on substance amounts, the total OTA and OTα excretion through feces constituted 18.70 ± 0.04% of the total intake. Consequently, the absorption rate of OTA and OTα in lactating sows was calculated to be 81.30 ± 0.04%. Additionally, the total amount of OTA and OTα excreted in the urine accounted for 8.40 ± 0.002% of the total intake.

## 3. Discussion

Research on the toxicokinetics of orally administered OTA has not been previously conducted in lactating sows. Thus, the primary objective of this study was to bridge the existing knowledge gap by investigating the entry rate of OTA into the body, as well as its absorption, distribution, metabolism, and elimination.

In our investigation into the toxicokinetics of OTA across various species, distinct patterns emerged concerning Tmax and T_1/2_Elim [24]. For lactating sows, Tmax occurred at 9 h, and T_1/2_Elim at 78.47 h. These findings indicate a longer Tmax in lactating sows compared to poultry [18,25], yet shorter than that observed in growing pigs [18]. Moreover, the T_1/2_Elim in lactating sows was found to be slightly shorter than in growing pigs [18], but significantly longer than in poultry species like broiler chickens and laying hens [25], as detailed in Table S1. The variability in OTA elimination half-life across species is likely due to pronounced differences in plasma protein affinity.

Regarding peak concentration (Cmax), a critical indicator of absorption and safety, we observed species-specific and administration route variations. In our study, lactating sows exhibited a Cmax of 0.92 μg/mL following a 500 μg/kg BW dose, a figure that aligns closely with that reported for growing pigs (1.74 μg/mL at 500 μg/kg BW), yet markedly lower than that observed in donkeys, as shown in Table S1. Notably, within the same species, intravenous injection yielded a higher Cmax than oral administration, underscoring the influence of administration route, dosage, and the physiological stage of the animals. Compared with other livestock animals, our research suggests that lactating sows exhibit characteristics of high absorption and slow excretion of OTA.

After OTA is absorbed, it is distributed throughout the body via the bloodstream, reaching various tissues and organs. This distribution, reflected by the volume of distribution (Vd) values detailed in Table S1, varies among species. In lactating sows, we observed a Vd nearly fourfold higher than in growing pigs, indicating a broader OTA distribution in these mammals. Our research reveals milk secretion as an additional route of OTA elimination in lactating sows, with peak OTA concentrations in milk occurring at 24 h post ingestion.

Table 5 compares our findings with previous research on growing pigs, indicating a similar rate of OTA excretion in urine; however, the proportion of OTα in our fecal excretion data is relatively low. This difference may be due to methodological limitations in analyzing fecal OTA metabolites, highlighting the significance of milk as a potential route for OTA elimination in our results. It is important to note that challenges in collecting milk samples, influenced by animal welfare considerations, could contribute to these variations. Furthermore, these differences are likely to impact the observed variability in absorption rates among pigs at different physiological stages.

This study marks the first identification of OTA residues in the milk of lactating sows. This finding is significant considering that OTA residues have previously been identified in the milk of dairy cows [26], dairy ewes [6], and in humans [27]. We observed that the milk-to-plasma (M/P) ratio increased from 0.06 to 0.46 within the first 24 h after ingestion (Table 6), indicating a dynamic pattern of lactational transfer. The ratio, although generally higher than those recorded in dairy ewes (M/P 0.04 to 0.21) over longer periods [6], aligns with human studies showing that dietary OTA contaminants can be transferred to breast milk [27]. The average OTA concentration in human breast milk represents about a quarter of that in plasma (M/P 0.25), with a notable increase in OTA excretion during the first week post delivery (M/P 0.4) [27].

Additionally, we must acknowledge the limitations of this study, such as significant fluctuations in OTA excretion in milk over time, necessitating that the M/P value may be viewed only as an approximate indicator of this mycotoxin’s transmission through milk.

## 4. Conclusions

In conclusion, this study is the first to determine the toxicokinetics of OTA in lactating sows, with the oral administration of 500 μg/kg BW. The peak plasma concentration of OTA was observed at 9 h. The maximum concentration of OTA in milk was noted at 24 h, while peak excretion in feces and urine occurred at 60 h and 54 h, respectively. Up to 120 h post administration, the cumulative excretion rates of OTA and OTα in feces and urine were 18.70% and 8.40%, respectively (calculated based on substance amounts). Notably, the elimination half-life of OTA in plasma was determined to be 78.47 h. The half-life of its metabolites was shorter, only 33.61 h for OTα. Additionally, a certain proportion of circulating OTA was excreted (M/P 0.06–0.46) within the first 24 h after OTA ingestion. These findings indicate that lactating sows have a high rate of OTA absorption and a slow excretion profile, and highlight the importance of considering milk as a significant elimination route for this mycotoxin. Further research is warranted to explore the potential impacts on their offspring.

## 5. Materials and Methods

### 5.1. Mycotoxins

The standards of OTA and OTα used for animal experiments and sample analysis in this study were obtained from Pribolab Biological Engineering Co. Ltd. (Qingdao, China), while acetonitrile (ACN) and methanol (MeOH) used for the sample analysis were all HPLC-MS grade (Fisher Chemical, Pittsburgh, PA, USA). Physiological saline and dimethyl sulfoxide (DMSO, sigma, Tokyo, Japan) used for the animal oral administration were both cell-culture grade.

### 5.2. Animals and Experimental Diets

The experiment was approved by the Animal Welfare and Ethics Committee of China Agricultural University. The feeding experiment was carried out at the Experimental Feeding Assessment Station of China Agricultural University.

Four healthy primiparous lactating sows of the French Large White breed, weighing 186.25 ± 10.31 kg, were selected and housed in metabolism cages, which were designed to ensure the collection all of fecal and urine samples. The blank blood, feces, urine, and milk samples were obtained 4 h before the beginning of the experimental dosing. During both the pre-experimental and the experimental phases, the sows were fed a customized lactating sow diet (Table 7), which was confirmed to have undetectable levels of Ochratoxin A (OTA). The detected concentrations of Aflatoxin B1 (AFB1), Zearalenone (ZEN), and Deoxynivalenol (DON) were 0.85, 4.3, and 27.8 μg/kg, respectively.

### 5.3. Toxin Administration Route

The *Aspergillus ochraceus* corn culture, containing 640 mg/kg of OTA, was the same batch sample used by Kang et al. (2023) [22]. The amounts of culture material given orally to the sows contained a single OTA dose of 500 µg/kg BW, which equals the OTA dose previously used by Galtier et al. in their pig study [18]. In detail, according to the OTA dose of 500 µg/kg BW, 137–152 g culture (containing OTA 87.5–97.5 mg) was accurately weighed into a plastic beaker and then dissolved and stirred in 100 mL of distilled water to form a suspension. Subsequently, this suspension was rapidly introduced into the stomachs of the lactating sows through an esophageal tube.

### 5.4. Collection of Plasma, Feces, Urine, and Milk

The blood samples were obtained from the anterior vena cava via indwelling needles in sows before administration (0 min) and a 5, 15, and 30 min, and 1, 2, 3, 6, 9, 12, 24, 48, 72, 88, 96, and 120 h after OTA post administration. The samples stored in heparin anticoagulation tubes were transferred to the laboratory, and then centrifuged at 3000 rpm for 15 min to obtain plasma. Moreover, the feces were collected before administration (0 h) and at 6, 12, 24, 36, 48, 60, 72, 84, 96, 108, and 120 h post administration. Urine samples were collected before administration (0 h) and at 6, 12, 18, 24, 30, 36, 42, 48, 54, 60, 66, 72, 78, 84, 90, 96, 102, 108, 114, and 120 h following administration. Milk samples were collected at 0, 6, 12, 24, 36, 48, 72, 96, and 120 h after the administration. Meanwhile, the feces weight and urine volume were recorded at each collection time. All samples were stored at −20 °C for further analysis.

### 5.5. Standard Solutions

Obtained from Pribolab (Qingdao, China), the stock solution of 1 mg/mL OTA and OTα was subsequently diluted with 50% acetonitrile to generate diverse concentrations of OTA and OTα working standard solutions (0.0125, 0.025, 0.05, 0.125, 0.25, 0.5, 1, 2.5, and 5 micrograms per milliliter). For the preparation of calibration samples, blank plasma, urine, and milk samples, each with a volume of 90 μL, were utilized. To these, adding 10 μL of the diverse concentrations of the working solutions yielded spiked samples at nine different concentration levels, ranging from 1.25 to 500 μg/L. Similarly, in the case of fecal samples, 1 g of blank material was employed, along with the addition of 100 μL of different concentrations of the working standard solution, leading to nine spiked sample concentration levels in the identical range. These spiked plasma, urine, milk, and fecal samples were then processed and analyzed using established treatment and detection methods specific to each sample type.

### 5.6. Sample Pretreatment

For plasma, urine, and milk, after thawing, a 1 mL sample was transferred to a 10 mL centrifuge tube, and 300 μL of 0.2 M sodium sulfate solution, 400 μL of 0.2 M acetate buffer (pH 5.5), and 300 μL of the enzyme solution were added. After thorough vortex mixing, the mixture was incubated at 37 °C in a water bath overnight (17 h). Then, 4 mL of acetonitrile/water (80/20, *v*/*v*) was added. Among them, the enzyme solution was prepared by dissolving 50 mg of the H-1 type β-glucuronidase (3,023,000 units/g) in 10 mL of 0.2 M acetic acid buffer (pH 5.5).

For feces, after drying and homogenization, 2 g was placed in a 50 mL stoppered plastic centrifuge tube, and 20 mL of acetonitrile/water (80/20, *v*/*v*) was added.

On this basis, the mixture was vortexed for 2 min, sonicated for 1 h, and centrifuged at a speed of 8000 rpm/min for 7 min. Then, 1 mL of supernatant was taken from the centrifuged feces, urine, plasma, and milk, respectively, it was filtered using a 0.22 μm filter membrane for further analysis.

### 5.7. UPLC-MS/MS Method Validation

During method validation, linearity, sensitivity, and recovery were assessed individually for plasma, urine, feces, and milk samples. Calibration curves were generated using four separate blank matrices, encompassing plasma, urine, feces, and milk, across 9 concentration levels ranging from 1.25 to 500 μg/L. Sensitivity was evaluated by determining the limit of detection (LOD) and limit of quantification (LOQ), with the LOD achieving a signal-to-noise (S/N) ratio of ≥3, and the LOQ reaching a S/N ratio of ≥10. To assess the method’s accuracy in different matrices, recovery efficiency was evaluated by comparing the peak area of two OTA and OTα concentrations (20 and 100 μg/L) in the spiked samples to the OTA and OTα peak area in the corresponding standard working solutions.

### 5.8. Detecting OTA and OTα in Plasma, Feces, Urine, and Milk Using UPLC-MS/MS

UPLC was performed on a Waters Acquity (Milford, MA, USA) system. Chromatographic separation was achieved on an ACQUITY UPLC BEH C18 (100 mm × 2.1 mm, 1.7 μm) column (Waters, Milford, MA, USA). The flow rate was 0.3 mL/min and the injection volume was 2 μL. The mobile phase was consisted of 0.1% formic acid in water (A) and methanol (B). A linear gradient elution program was applied as follows: 0–2 min 5% B; 2–3 min 35% B; 3–6 min 65% B; 6–10 min 65% B; 10–12 min 95% B; and 12–13.5 min 5% B (total run 15 min). Mass spectrometry analysis was carried out using an A 5500 triple-quadrupole tandem mass spectrometer (AB Sciex, Framingham, MA, USA) equipped with an electrospray interface (ESI) operating in positive mode for OTA. The ESI conditions were as follows: ion spray voltage (IS) at 5.5 kv; curtain gas (CUR) at 20 psi; nebulizer gas (GS1) at 55 psi; and ion source temperature at 450 °C. The mass spectrometry conditions are presented in Table S2.

### 5.9. Statistical Analysis

Statistical analysis was performed based on the standardized concentrations of OTA and OTα in plasma, urine, feces, and milk. Plasma toxicokinetic parameters of OTA and OTα were calculated using the non-compartmental modeling in WinNonlin 5.2.1 software (Certara, Inc., Princeton, NJ, USA). Then, the average OTA and OTα concentrations in plasma and milk at different times were used to chart the plasma and milk concentration–time profiles. The mean excretions of OTA and OTα in feces and urine at different times were used to plot the OTA and OTα excretion–time profiles. The figures were charted using GraphPad Prism version 9.4.1 (GraphPad Software, Inc., San Diego, CA, USA). Data are shown as mean ± SEM.

## Figures and Tables

**Table 1:** Calibration plots for OTA and OTα in plasma, feces, urine, and milk.

Matrix	Composition	Slope	*R* ^2^	Range (µg/L)/(µg/kg)	Sensitivity (µg/L)/(µg/kg)	
					LOD	LOQ
Plasma	OTA	12,154	0.9988	1.25–500	0.2	0.6
	OTα	12,048	0.9994	1.25–500	0.2	0.6
Feces	OTA	11,718	0.9963	1.25–500	0.4	1.2
	OTα	11,531	0.9972	1.25–500	0.4	1.2
Urine	OTA	4219	0.9922	1.25–500	0.2	1.0
	OTα	4316	0.9954	1.25–500	0.2	1.0
Milk	OTA	13,436	0.9993	1.25–500	0.2	0.6
	OTα	13,444	0.9997	1.25–500	0.2	0.6

OTA and OTα concentrations in plasma, urine, and milk are measured in μg/L, while OTA and OTα concentrations in feces are expressed in μg/kg; *n* = 3 of each concentration.

**Table 2:** Recovery rates of OTA and OTα in plasma, feces, urine, and milk.

Item	Spike Level (µg/L)/(µg/kg)	Average Recovery (%)			
		Plasma	Feces	Urine	Milk
OTA	20	87.03	85.50	90.68	96.65
	100	84.25	82.50	82.30	92.78
OTα	20	86.95	87.20	92.35	95.20
	100	85.23	83.60	84.50	93.60

*n* = 3 of each concentration; µg/L refers to the values of plasma, urine and milk; µg/kg refers to the values of feces.

**Table 3:** Toxicokinetic characteristics of OTA and OTα in plasma following a single oral intake of OTA in lactating sows.

Parameters	Value of OTA	Value of OTα
OTA (μg/kg BW)	500	
Tmax (h)	9.00 ± 0.00	12.00 ± 0.00
Cmax (µg·L^−1^)	920.25 ± 88.46	3.45 ± 0.07
T_1/2_Elim (h)	78.47 ± 7.68	33.61 ± 12.13
AUC (µg·mL^−1^·h)	69.50 ± 7.90	0.21 ± 0.01
MRT (h)	107.78 ± 9.33	51.91 ± 11.98
Cl (L·kg·BW^−1^·h^−1^)	0.0014 ± 0.00	0.44 ± 0.02
Vd (L/kg BW)	0.16 ± 0.003	26.18 ± 13.36

Tmax: time at maximum concentration of OTA and OTα in plasma; Cmax: maximum concentration of OTA and OTα in plasma; T_1/2_Elim: terminal elimination half-life; AUC: area under plasma concentration–time curve; MRT: mean residence time; Cl: total plasma clearance; Vd: volume of distribution.

**Table 4:** Amount of OTA and OTα in both feces and urine in lactating sows.

Parameters	Mass Value (mg)	The Amount of Substance (µmol)
Body weight (kg)	186.25 ± 10.30	
OTA (μg/kg BW)	500	
OTA intake	93.12 ± 5.15	230.62 ± 12.76
OTA excretion via feces	0.07 ± 0.01	0.17 ± 0.02
OTα excretion via feces	11.02 ± 2.38	42.96 ± 9.28
Total OTA and OTα excretion through feces(%)	11.91 ± 0.03	18.70 ± 0.04
Absorption rate (%)	88.09 ± 0.03	81.30 ± 0.04
OTA excretion via urine	2.46 ± 0.18	6.11 ± 0.47
OTα excretion via urine	3.40 ± 0.04	13.26 ± 0.18
Total OTA and OTα excretion through urine (%)	6.29 ± 0.001	8.40 ± 0.002

OTA and OTα excretion through feces (%) = OTA and OTα excretion via feces/OTA intake × 100. OTA and OTα excretion through urine (%) = OTA and OTα excretion via urine/OTA intake × 100. Absorption rate (%) = (OTA intake − total OTA and OTα excretion through feces)/OTA intake × 100.

**Table 5:** Proportional excretion of OTA and its corresponding metabolite ochratoxin α in the matrices of pigs.

Parameters	Value	Value
OTA (μg/kg BW)	500	66 *
OTA excretion via feces (%)	0.08 ± 0.00	0.8 ± 0.2
OTα excretion via feces (%)	18.63 ± 0.04	45.4 ± 2.7
OTA excretion via urine (%)	2.65 ± 0.002	5.5 ± 1.9
OTα excretion via urine (%)	5.75 ± 0.001	3.8 ± 0.3
Animals		
Physiological stage	Lactating	Growing
Body weight (kg)	186.25 ± 10.30	36.7 ± 2.0
Reference	This study	Blank et al. (2005) [28]

* (1) Co-contamination of the feed sample for all animals, not only OTA. (2) The animals were given a single dose of 150 g wheat containing with 2.42 mg OTA, 0.36 mg OTB, 0.03 mg Otα, and 0.1 mg OTβ, which converted to 65.9, 9.8, 0.8, and 2.7 μg/kg BW by the authors of this study to compare on the same basis.

**Table 6:** The milk-to-plasma (M/P) ratio of OTA at different time points in lactating sows.

Time	Milk Concentration of OTA (μg/L)	Plasma Concentration of OTA (μg/L)	* M/P
0 min	0	0	0
6 h	39.55 ± 14.77	792.30 ± 84.57	0.06 ± 0.01
12 h	137.87 ± 3.67	681.40 ± 15.98	0.20 ± 0.01
24 h	200.03 ± 35.35	472.50 ± 25.88	0.46 ± 0.08
48 h	69.35 ± 9.34	368.60 ± 11.46	0.18 ± 0.04
72 h	0	308.85 ± 26.80	0

* M/P = Milk concentration of OTA/Plasma concentration of OTA.

**Table 7:** Composition and nutrient levels of basal diet %.

Ingredients	Content	Nutrient Levels ^②^	Content
Corn	55.50	Net energy (NE) ^③^	10.67
Soybean meal	13.00	Crude Protein (CP)	17.50
Steamed dried fish meal	2.00	Calcium (Ca)	0.96
Extruded soybean	7.00	Total phosphorus (TP)	0.65
Rice bran meal	7.00	Available phosphorus(AP)	0.36
Sugar beet pulp	2.00	Crude fiber (CF)	3.20
Coarse wheat bran	3.00	Sodium chloride (NaCl)	0.75
Extruded flax seed	2.50	Sodium (Na^+^)	0.21
Soybean oil	2.00	Chloride (Cl^−^)	0.43
Glucose	2.00	Standard ileal digestible lysine (SID-Lys)	1.10
Premix ^①^	4.00		

^①^ The premix for lactation sows provided the following per kilogram of diet: Ca (as calcium carbonate and calcium hydrogen phosphate) 7.5 g, TP (as calcium hydrogen phosphate) 1.9 g, NaCl (as sodium chloride) 6 g, SID-Lys (as L-lysine hydrochloride) 3.2 g, Fe (as ferrous sulfate) 116 mg, Cu (as copper sulfate) 25 mg, Zn (as zinc sulfate) 100 mg, Mn (as manganese sulfate) 48 mg, I (as calcium iodate) 1 mg, Se (as sodium selenite) 0.3 mg, VA 7000 IU, VD_3_ 3480 IU, VE 100 IU, VK_3_ 3 mg, VB_1_ 2 mg, VB_2_ 7.8 mg, VB_6_ 4 mg, VB_12_ 0.03 mg, Folic acid 2.5 mg, Nicotinic acid 33.25 mg, Pantothenic acid 15 mg, and Biotin 0.35 mg. ^②^ CP, CF, Ca, TP, and NaCl levels are measured values, while the other nutrient levels are calculated values. ^③^ The unit of net energy is MJ·kg^−1^.

## Data Availability

The datasets used and analyzed during the current study are available from the corresponding author upon reasonable request.

## Supplementary Materials

The following supporting information can be downloaded at: https://www.mdpi.com/article/10.3390/toxins16030128/s1, Table S1: Toxicokinetic Parameters Following Oral and Intravenous Administration in Various Animal Species. Table S2: Mass Spectrometry Conditions.
