# Supplementary material for: The Toxicokinetics, Excretion Patterns, and Milk Transmission of Ochratoxin A in Lactating Sows
Source: Toxins (Basel). 2024 Mar 1;16(3):128. doi: 10.3390/toxins16030128 (PMC10974951; doi:10.3390/toxins16030128)
Supplement: Supplementary file 1 [file toxins-16-00128-s001.zip › toxins-2831027-supplementary.pdf]

Table S1. Toxicokinetic parameters following oral and intravenous administration in various animal species.

Species	Route*	BW(kg)	Dose(mg/kg)	T_{max}(h)	C_{max}(μg/mL)	$T_{1/2}$Elim(h)	V_d(mL/kg)	CL(mL/kg)	Reference
Male Wistar rat	po	0.25-0.30	0.05	—	0.39	120	160	0.91	Hagelberg et al. (1989)[24]
Mouse	po	0.02	0.05	—	0.37	39	340	6.1	Hagelberg et al. (1989)[24]
Fish	po	1.00	0.05	—	0.014	0.68	57	58	Hagelberg et al. (1989)[24]
Quail	po	0.16	0.05		0.26	6.7	550	57	Hagelberg et al. (1989)[24]
Broiler chicken	po	1.27	0.25	1.43-4.63	0.05-0.08	8.2-14.11	270	710-770	Devreese et al. (2018)[25]
Leghorn chicken	po	1.84	2.0	0.33	0.78	4.15	2160	—	Galtier et al.(1981)[18]
Laying hen	po	2.05	0.25	0.75-1.88	0.05-0.06	17.3-21.15	230	890-1030	Devreese et al. (2018)[25]
Turkey	po	1.71	0.25	0.75-0.81	0.18-0.20	9.85-15.5	180	330-390	Devreese et al. (2018)[25]
Muscovy duck	po	2.68	0.25	0.31	0.04-0.05	35.14-39.02	760	430-610	Devreese et al. (2018)[25]
Rabbit	po	2-3	2.0	1	2.13	8.25	453	—	Galtier et al.(1981)[18]
Rhesus monkey	po	5.00	0.05	—	0.50	510	130	0.18	Hagelberg et al.(1989)[24]
Donkey	po	123.60	2.50	12	10.34	24.52	150	4.10	Kang et al. (2023)[22]
Growing pig	po	35	0.50	10	1.74	88.80	42.9	—	Galtier et al.(1981)[18]
Lactating sow	po	186.25	0.50	9	0.92	78.47	160	1.40	This study
Mouse	iv	0.02	0.05	—	0.37	48	420	6.1	Hagelberg et al. (1989)[24]
Male Wistar rat	iv	0.25-0.30	0.05	—	2.1	170	230	0.92	Hagelberg et al. (1989)[24]
Rat	iv	0.30	0.33	—	2.00	103	—	3.11	Li et al. (1997)[19]
Fish	iv	1.00	0.05	—	0.20	8.3	690	57	Hagelberg et al. (1989)[24]
Quail	iv	0.16	0.05	—	0.59	12	1500	86	Hagelberg et al. (1989)[24]
Broiler chicken	iv	1.27	0.25	—	0.91-1.20	22.2-23.95	270	610-740	Devreese et al. (2018)[25]
Leghorn chicken	iv	1.84	2.0	—	—	3.00	—	—	Galtier et al.(1981)[18]
Laying hen	iv	2.05	0.25	—	1.10-1.44	12.15-14.21	230	820-980	Devreese et al. (2018)[25]
Turkey	iv	1.71	0.25	—	1.43-1.48	11.28-18.19	180	290-380	Devreese et al. (2018)[25]

Muscovy duck	iv	2.68	0.25	—	0.31-0.66	16.76-17.04	760	710-740	Devreese et al. (2018)[25]
Rabbit	iv	2-3	2.0	—	—	10.8	—	—	Galtier et al.(1981)[18]
Vervet monkey	iv	2.40	0.8,1.5,2.0	2	26.50	484	118	0.22	Stander et al.(2001)[20]
Rhesus monkey	iv	5.00	0.05	—	0.98	840	200	0.17	Hagelberg et al.(1989)[24]
Growing pig	iv	35	0.50	—	—	84.5	—	—	Galtier et al.(1981)[18]

*Administration route: po refers to oral administration , iv refers to injection.

Table S2: Mass spectrometry conditions

Mycotoxin	Molecular Formula	RT/min	MRM（m/z）	Declustering Potential(DP)	Collision Energy(CE)
OTA	$C_{20}H_{18}ClNO_6$	8.28	404.2/239.2*	120	33
			404.2/358.3	150	18.7
OTα	$C_{11}H_9ClO_5$	6.13	255.1/167.0*	-55	-33
			255.1/211.0	-36	-21

*Transitions used for quantification.
